# A Perfluorocarbon-Coated ZrP Cation Exchanger with Excellent Ammonium Selectivity and Chemical Stability: An Oral Sorbent for End-Stage Kidney Disease (ESKD)

**DOI:** 10.1021/acs.langmuir.3c00753

**Published:** 2023-05-25

**Authors:** Evan Richards, Sang-Ho Ye, Stephen R. Ash, Lei Li

**Affiliations:** † Department of Chemical and Petroleum Engineering, University of Pittsburgh, Pittsburgh, Pennsylvania 15260, United States; ‡ McGowan Institute for Regenerative Medicine, Pittsburgh, Pennsylvania 15219, United States; § Department of Surgery, University of Pittsburgh, Pittsburgh, Pennsylvania 15260, United States; ∥ CEO, HemoCleanse Technologies, LLC, Lafayette, Indiana 47904, United States

## Abstract

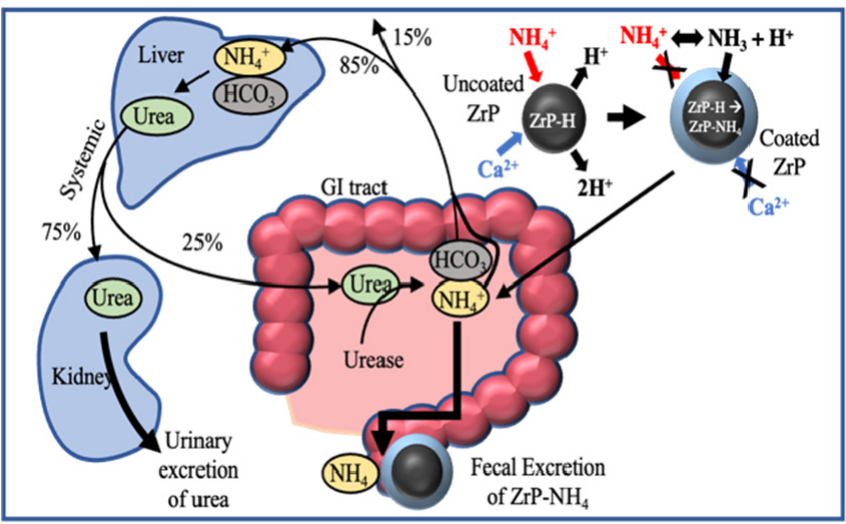

An oral sorbent to remove NH_4_^+^ within the small intestine of end-stage kidney disease (ESKD) patients could reduce blood urea levels and diminish their dialysis treatment burden. But current sorbent materials like amorphous zirconium phosphate particles Zr(HPO_4_)_2_·H_2_O (ZrP) lack the selectivity to remove NH_4_^+^ in water solution with other competing ions. Our previous work found that a gas-permeable, hydrophobic polydimethylsiloxane (PDMS) coating on ZrP improved the material’s selectivity for NH_4_^+^. However, a competing ion Ca^2+^ was still removed by PDMS-coated ZrP sorbent, and the permeability of the PDMS coating to Ca^2+^ was increased after low-pH stomach-like condition exposure. An alternative hydrophobic and gas permeable coating has been investigated—perfluorooctyltriethoxysilane (FOTS). The coating was attached in place of PDMS to a tetraethyl orthosilicate-coated ZrP surface. Surface atomic composition analysis and scanning electron microscopy observation verified the successful application of the FOTS coating. Water contact angle analysis validated the FOTS coating was hydrophobic (145.0 ± 3.2°). *In vitro* competing ion studies indicated the FOTS coating attached to ZrP increased NH_4_^+^ removal by 53% versus uncoated ZrP. FOTS offers complete selectivity for NH_4_^+^ over Ca^2+^ with similar NH_4_^+^ capacity as the previous PDMS coating. Moreover, FOTS-coated ZrP maintained NH_4_^+^ removal capacity and selectivity after the acid exposure study, indicating excellent acid resistance while NH_4_^+^ selectivity of ZrP-PDMS decreased by 72%. The results suggested that FOTS-coated ZrP is promising as an oral sorbent for ESKD patients.

## Introduction

Approximately 37 million people within the United States are estimated to have chronic kidney disease (CKD).^1^ Over 131000 people in the United States alone started treatment for end-stage kidney disease (ESKD) in 2018.^1^ Nearly 786000 people in the United States are currently living with ESKD.^1^ Peritoneal dialysis aims to mitigate the time requirement faced by ESKD patients on hemodialysis by allowing them to perform dialysis away from the clinic and on a more continuous schedule. But overall toxic uremic retention solutes removal is less efficient for peritoneal dialysis than for hemodialysis with the clinician not present.^2^ The higher uremic retention solute levels, mechanical issues, and infections often associated with peritoneal dialysis ultimately lead to many patients changing from peritoneal dialysis to hemodialysis.^3^ The quality of life for patients receiving hemodialysis or peritoneal dialysis is poor^4^ due to high treatment burden,^2^ increased risk of infections,^2^ and dietary restrictions.^5^ An improved ESKD treatment process could benefit both the patient and clinician.

One of the methods to improve a patient’s quality of life is by way of a wearable or highly portable artificial kidney. The “REDY sorbent system” was a portable artificial kidney used from 1973 to 1994.^6−9^ Zirconium phosphate (ZrP, a nonselective cation exchanger) was used in the REDY sorbent system to capture NH_4_^+^ generated by urease action on urea. But the exchanger’s nonselectivity in the presence of other ions limited its binding capacity to approximately 0.95 mequiv of NH_4_^+^/g of ZrP.^9^ Up to 1 kg of ZrP was used within the REDY sorbent column. ZrP’s nonselectivity lowered Ca^2+^, Mg^2+^, and K^+^ levels in the dialysate. These ions were replenished by infusing them back into the dialysate.^9,10^ The REDY column also released counterions such as Na^+^, caused pH changes, and released NH_4_^+^ when saturated. Several research groups have developed techniques to counter these problems, but these have increased the complexity of the sorbent system.^9−11^

Oral sorbents have been clinically prescribed in CKD to mitigate the accumulation of toxic uremic retention solutes between dialysis treatments. The CKD-related uremic retention solutes capable of being clinically controlled are phosphate,^12−14^ potassium,^15,16^ cholesterol,^17^ and hydrogen ions.^18,19^ However, no oral sorbents have been developed for removing accumulated Na^+^ or urea from ESKD patients in between dialysis treatments.^9,10,20−22^ A coated ZrP sorbent with a high capacity for NH_4_^+^ could be used to alleviate patients before starting dialysis as their diets are modified to avoid protein consumption that causes urea accumulation.^23^ The coated ZrP sorbent would be given in combination with other cation exchangers to remove Na^+^ and K^+^ and the anion exchanger zirconium oxide to control pH, phosphate, and other negatively charged toxic uremic solutes like oxalate. The treatment could even delay dialysis for an ESKD patient. Our previous work demonstrated that a nonselective cation exchanger like hydrogen (H^+^) loaded ZrP could be coated with a gas-permeable and hydrophobic membrane to improve its binding capacity for NH_4_^+^ and selectivity in the presence of other ions.^24^ The hydrophobic coating served as a barrier to ions in water solution interacting with the cation exchanger. The coating’s gas permeable property allowed for gaseous ammonia (NH_3_) transfer to the H^+^-loaded exchanger. As a gas, NH_3_ could pass through the membrane, bind with H^+^ to form NH_4_^+^, and thus be “trapped” within the capsule. For the “acid trap” to work,^25^ the membrane needed to be impermeable to liquid water because water channels could also allow transfer of various cations in solution. A selective sorbent material would have increased binding capacity for NH_4_^+^ in the small intestine of ESKD patients. And a sorbent material selective for NH_4_^+^ that is also capable of being ingested might postpone the necessity for dialysis treatment in ESKD patients.

Our previous work found that polydimethylsiloxane (PDMS)-coated ZrP improved the material’s selectivity for NH_4_^+^ by nearly 4-fold, which has been attributed to the coating’s gas-permeable and hydrophobic nature. The hydrophobic PDMS coating prevents ions in water solution from reaching the ion exchanger while allowing gaseous ammonia transfer to ZrP. Simulated small intestine studies found that PDMS-coated ZrP removed 94 ± 11% more NH_4_^+^ than uncoated ZrP with Ca^2+^ as the competing ion.^24^ Ca^2+^ removal also decreased by 64 ± 6%.^24^ The nearly 300% increase in selectivity for NH_4_^+^ was attributed to the gas-permeable and hydrophobic coating on the surface of ZrP. But the coating needed improvement as Ca^2+^ was still removed to some extent from solution by the sorbent.

A more ordered PFC-based structure on ZrP’s surface could improve both NH_4_^+^ selectivity and removal capacity in the presence of other competing ions. A PFC-based coating could also maintain its capacity and selectivity for NH_4_^+^ after exposure to HCl_(aq)_ due to improved chemical stability versus PDMS-based coatings. PFC-based materials are ideal candidates due to their highly ordered packing structure, increased hydrophobicity,^26^ good gas permeability,^27,28^ and continued usage within the medical industry for devices,^27,28^ surgical procedures,^29−31^ and medical imaging methods.^32^ 1*H*,1*H*,2*H*,2*H*-Perfluorooctyltriethoxysilane (FOTS) was the selected PFC-based material used in this work. *In vitro* experiments compared the effect of FOTS-coated ZrP materials with the previously studied PDMS-coated material.^24^ We coated the TEOS-based surface of ZrP with FOTS. We varied the concentration of FOTS to optimize the coating property and compared the coatings via a scanning electron microscopy (SEM), X-ray photoelectron spectrometry (XPS), and competing ion binding study before and after acid treatment. The acid stability of the FOTS-coated ZrP was also compared to PDMS-coated ZrP.

## Materials and Methods

### Materials

Amorphous ZrP in granular form (39 ± 3 μm) was provided by HemoCleanse Technologies LLC (Lafayette, IN), loaded almost entirely with hydrogen (but with some residual sodium). Hydrochloric acid (CAS No.: 7647-01-0), HEPES sodium salt (CAS No.: 75277-39-3), acetone (CAS No.: 67-64-1), calcium chloride (CAS No.: 10043-52-4), and ammonium chloride (CAS No.: 12125-02-9) were purchased from Sigma-Aldrich (St. Louis, MO). Tetraethyl orthosilicate (CAS No.: 78-10-4) was purchased from Ge.leste, Inc. (Morrisville, PA). 1*H*,1*H*,2*H*,2*H*-Perfluorooctyltriethoxysilane, 97% (CAS No.: 51851-37-7), was purchased from Fisher Scientific (Waltham, MA). Urea nitrogen used to measure total of NH_4_^+^ and NH_3_ (CAS No.: B551-132) and calcium (CAS No.: C503-480) colorimetric testing kits were purchased from Teco Diagnostics (Anaheim, CA). All water in experiments was deionized (DI). Colorimetric testing kits from Teco Diagnostics were used to quantify NH_4_^+^ and Ca^2+^ levels in solution. Color development assays were prepared according to the instructions given with each kit. [NH_4_^+^] and [Ca^2+^] were quantified using a Genesys S10 UV–vis monochromator in Dr. Haitao Liu’s Laboratory at University of Pittsburgh. The monochromator was set to wavelengths 570 nm (blue/green) and 630 nm (violet) for Ca^2+^ and NH_4_^+^ measurements, respectively. 3 mL cuvettes used for the studies were purchased from Cole-Parmer with a 10 mm path length (CAS No.: 759075D). 4.5 mL cuvettes used for the studies were purchased from Fisher Scientific with a 10 mm path length (CAS No.: S29159).

### ZrP Coating and Characterizations

Amorphous ZrP was coated with TEOS and FOTS using a standard silanization technique.^33^ 1 mL of TEOS and 1 mL of deionized (DI) water were added to a 50 mL Falcon tube. 20 mL of acetone was then poured into the solution to combine the two layers (pH = 5.5 ± 0.5). A 1 cm stir bar was placed inside the tube and the tube was capped. The capped Falcon tube was placed on a rocking panel for 2 h. Uncoated ZrP (1.00 ± 0.005 g) was added to the solution after mixing. The tube was capped and placed on a rocker panel at room temperature overnight at 30 rpm (pH = 5.5 ± 0.5). The Falcon tube was removed from the rocker panel and positioned upright in a test tube holder, undisturbed for 2 h. The effluent above the undisturbed ZrP bed was then removed with a disposable pipet, and ZrP was gently washed with acetone. The uncapped tube was placed inside a low-moisture chamber at room temperature for curing (72 h). The resulting product (ZrP-T) was washed with acetone and DI water and then placed in a Falcon tube.

Three separate batches of each of the five materials were made with varying concentrations of FOTS and DI water in 10 mL of acetone. The concentration of FOTS in acetone during the coating process was varied between 0.1% and 10% for different batches of modified ZrP to evaluate the impact that thickness of the PFC-based membrane could have on NH_4_^+^ total removal from water solution. The preparation and designated names are given in Table 1.

**Table 1:** Amount of DI Water and FOTS Added to 10 mL of Acetone to Form PFC-Coated ZrP Ranging from 0.1 to 10.0%^a^

material nomenclature	DI water added (μL)	FOTS material added (μL)	FOTS% of 10 mL solution
ZrP-FOTS_0.1%_	10	10	0.1
ZrP-FOTS_1.0%_	100	100	1.0
ZrP-FOTS_4.0%_	400	400	4.0
ZrP-FOTS_7.0%_	700	700	7.0
ZrP-FOTS_10.0%_	1000	1000	10.0

a The designated nomenclature is given for each material.

**Scheme 1:**
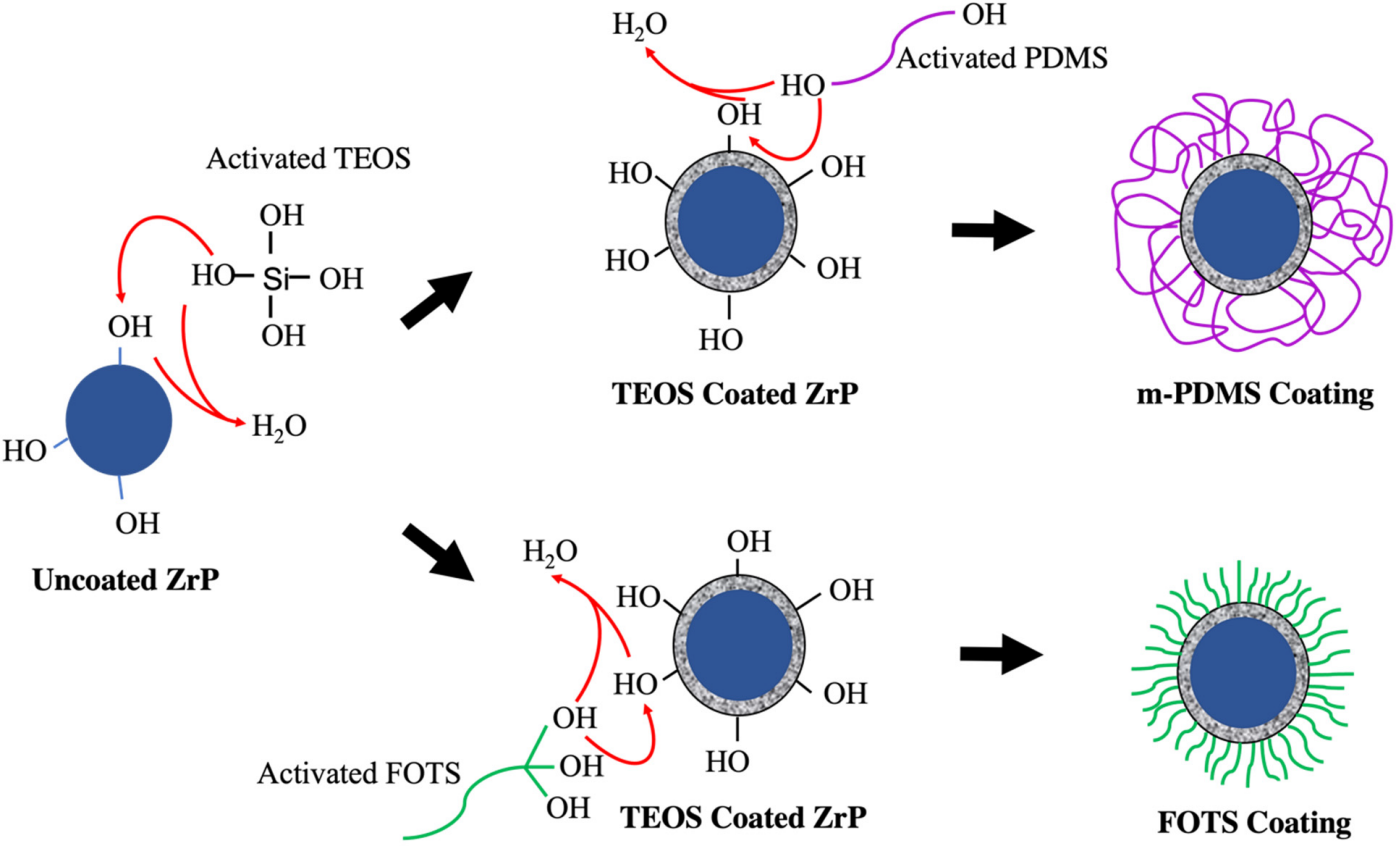
Hydrogen-Loaded ZrP Was First Coated with Activated TEOS to Form a Polysiloxane Membrane on the Exchanger’s Surface; the Polysiloxane Surface Was Then Coated with Either Activated PDMS (Top) or FOTS (Bottom) The FOTS coating yielded a more ordered and densely packed structure on the surface of ZrP. The wet chemistry used for all coatings was a straightforward silanization technique.

Designated amounts of FOTS and DI water from Table 1 were added to a Falcon tube for each of the five materials. 10 mL of acetone was added into the solution to combine the two layers (pH = 5.5 ± 0.5). The curing step used for TEOS membrane formation was repeated for FOTS surface attachment (ZrP-FOTS). Additional ZrP was then coated with 10% FOTS (twice) without first coating by TEOS (ZrP-F_2_) to compare to coatings with the TEOS layer. The PDMS coating protocol used in this work was the same as reported in our previous work.^24^ In brief, the polysiloxane layer was formed using TEOS as done with the FOTS-coated materials, above. m-PDMS (10% in solution) was attached to the polysiloxane surface via silanization and formed the gas-permeable and hydrophobic membrane. The m-PDMS coating protocol was then repeated a second time (ZrP-PDMS). Scheme 1 summarizes the coating process for the PDMS-based coating and FOTS-based coating.

The atomic surface composition of both PDMS- and FOTS-coated materials was determined via surface analysis using XPS at the University of Washington’s Molecular Analysis Facility. A detail scan was run for Zr, F, P, Si, and Na^+^ to improve quantification. Data analysis was performed using the Service Physics Hawk Analysis 7 program (Service Physics, Bend, OR). Each sample was pressed flat onto a piece of double-sided Scotch tape that was adhered to a clean silicon wafer. The samples were then adhered to the sample holder with double-sided Scotch tape. The samples were handled with solvent-cleaned tweezers while wearing polyethylene gloves. Three spots on each sample were chosen for analysis.

Scanning electron microscopy (SEM) images were collected for both PDMS- and FOTS-coated materials at the University of Pittsburgh’s Center for Biological Imaging using a JSM 6335F SEM (JEOL Ltd., Japan). The instrument had a magnification range from 10× up to 500000×. Particle sizes were measured and the heterogeneity was determined via a 100× magnification. The size was determined by measuring the horizontal and perpendicular diameters of the particles. The surfaces of the different ZrP coatings were assessed at 10000×.

The wettability of ZrP-PDMS, ZrP-FOTS_1%_, ZrP-FOTS_4%_, and ZrP-FOTS_7%_ was characterized in Dr. Lei Li’s Laboratory at the University of Pittsburgh via water contact angle (WCA) methods WCA_s_ (static), WCA_a_ (advancing), and WCA_r_ (receding). The materials were analyzed using a VCA Optima XE system (AST Products, Inc., Billerica, MA) at room temperature and 48% humidity. 50 mg samples of each material were pressed into pellets using a pellet press. The pellets formed from the material were then placed on the VCA instrument platform directly underneath a syringe filled with DI water. WCA_s_ data were collected by forming a 1–2 μL droplet on the tip of the syringe. The platform holding the pellet was then carefully elevated to the water droplet. The needle was then withdrawn to leave the droplet resting on the surface of the material. An image of the droplet was then taken using a charged-couple device camera, and the value of WCA_s_ was determined via VCA software. WCA_a_ analysis was performed by carefully placing the tip of the fluid-filled syringe back into the formed water droplet and slowly injecting additional fluid. Fluid was continuously injected into the droplet while maintaining the contact line until the WCA no longer increased. The WCA_a_ was defined to be the maximum WCA during injection. The instrument continually recorded as fluid was injected to determine the maximum angle. WCA_r_ was determined by gently pulling water back out of the droplet with the syringe. The droplet was again continually recorded to find the receding angle. Hysteresis was calculated as the difference between WCA_a_ and WCA_r_. All experiments were performed in triplicate with two angles reported at each measurement (*n* = 6).

### Competing Ion Study

This study was performed to determine each FOTS sorbent material’s capacity and selectivity for NH_4_^+^ in the presence of a competing ion (Ca^2+^). Ca^2+^ is a divalent cation with a much higher affinity for ZrP than NH_4_^+^, Mg^2+^, K^+^, or Na^+^. A previously published work defined a concentration of Ca^2+^ that had the same competition for binding on uncoated ZrP as occurred with a solution of Ca^2+^, Mg^2+^, K^+^, NH_4_^+^, and Na^+^ at the expected concentrations in the small intestine.^24^ Based on results of these studies, the solutions used in this work contained equimolar concentrations of Ca^2+^ and NH_4_^+^ (35 mM each).

The materials assessed in the current study were ZrP-FOTS_4%_ and ZrP-FOTS_7%_. FOTS-coated sorbent materials were studied versus uncoated ZrP and ZrP-PDMS. Three batches of ZrP-PDMS were developed to compare to FOTS-based coatings.^24^ A testing solution composed of 35 mM NH_4_^+^, 35 mM Ca^2+^, and 20 mM sodium based (4-(2-hydroxyethyl)-1-piperazineethanesulfonic acid) (HEPES) buffer was made for the study (starting pH = 8.3). Sodium-loaded HEPES was used within the experiments to buffer the alkaline pH during removal studies. HEPES is one of the “good” buffers commonly used within biological research due to its high level of water solubility, limited production of sodium, and p*K*_a_ value within the biological range 6–8.^34^

Briefly, 50 mg from each batch of material was placed in individual 24 mL test tubes (three test tubes for each material). 5 mL of testing solution was placed into each test tube, and the tubes were capped and immediately placed horizontally on an orbital shaker plate with a 3 cm radial axis. The shaker plate was then set to 270 rpm to ensure suspension of the dense ZrP particles in the solution. Each test tube was tested for [Ca^2+^] and [NH_4_^+^] via colorimetric analysis. [NH_4_^+^] data were collected at time points *t* = 0 min, 20 min, 40 min, 1 h, 2 h, 3 h, 6 h, and 24 h. [Ca^2+^] data were collected at *t* = 1, 2, 3, 6, and 24 h (assumed to be an average of gut transit time). All data points were collected in triplicate, each sample created in separate coating procedures. The selectivity was calculated as . The coated particles floated on the water due to their hydrophobic nature. A 10 μL pipet tip was lowered below the particles floating on the water surface during sampling to avoid removing sorbent particles in the sample. The uncoated ZrP particles sedimented quickly when stationary, creating a thin layer of clear fluid on top of the sorbent suspension. The pipet tip was placed into this layer of clear fluid to remove supernatant samples.

### Acid Exposure Study

A solution of DI water was set to a pH value of 1.8 using HCl_(aq)_. 20 mL of solution was poured into three 50 mL centrifuge tubes. 100 mg of ZrP-PDMS, ZrP-FOTS_4%_, and ZrP-FOTS_7%_ was added to all six 24 mL test tubes. The test tubes were capped and placed upright for 3 h. The acid solution was then carefully removed from the coated sorbent material via a disposable pipet. The sorbent was then carefully washed with 20 mL aliquots of DI water, three times each. The stability of the coatings was quantified by repeating the analytical and *in vitro* testing performed on the materials before acid exposure.

### Statistical Methods

All tests were performed in triplicate. Three batches of each material were made and individually tested. Experimental values were presented as the means ± standard deviation. Particle and indentation width distributions were found using 95% confidence interval. Mean, standard deviation, and confidence intervals (CI) were all calculated via Microsoft Excel. *P* values were calculated by using the standard *t* test.

## Results and Discussion

### Characteristics of PFC-Coated Materials

**Figure 1:**
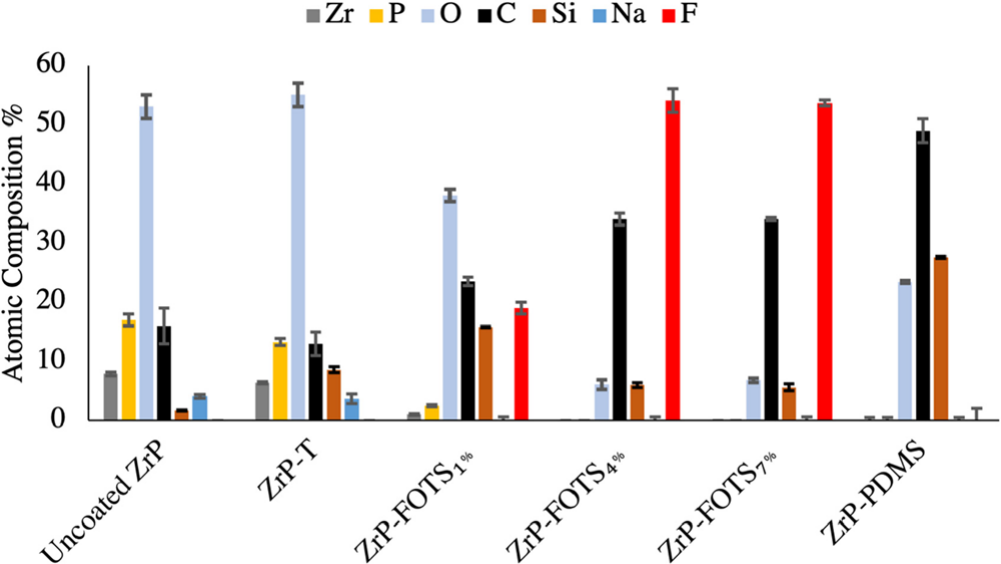
XPS indicated surface composition of ZrP and ZrP-T and membrane coatings on ZrP-FOTS_1%_, ZrP-FOTS_4%_, ZrP-FOTS_7%_, and ZrP-PDMS. Results indicate the PFC- and PDMS-based coatings were at least 10 nm thick for all coated materials except ZrP-FOTS_1%_. Each experiment was performed three times (*n* = 3). Carbon on uncoated ZrP could be from ethanol washes before XPS analysis.

XPS surface analysis results shown in Figure 1 include the atomic surface concentrations of uncoated ZrP, ZrP-FOTS_1%_, ZrP-FOTS_4%_, and ZrP-FOTS_7%_. The Zr and P concentrations on all coated surfaces decreased after coating applications. Decreased Zr and P indicated the addition of a coating to ZrP’s surface. ZrP-FOTS_1%_ had 50% less F on the surface than ZrP-FOTS_4%_ and ZrP-FOTS_7%_. ZrP-FOTS_1%_ surface concentration also had 6× more O than the other FOTS-coated materials. ZrP-FOTS_4%_ and ZrP-FOTS_7%_ results showed a shift toward the theoretical percentages of FOTS (F = 52%, C = 32%, Si = 4%, and O = 12%) from the atomic percentages of uncoated ZrP as the coating thickness increased. The FOTS coating was at least ∼10 nm thick for ZrP-FOTS_4%_ and ZrP-FOTS_7%_ because the takeoff angle was 90°, and XPS quantifies atoms to a coating depth of ∼10 nm beneath the surface.

**Figure 2:**
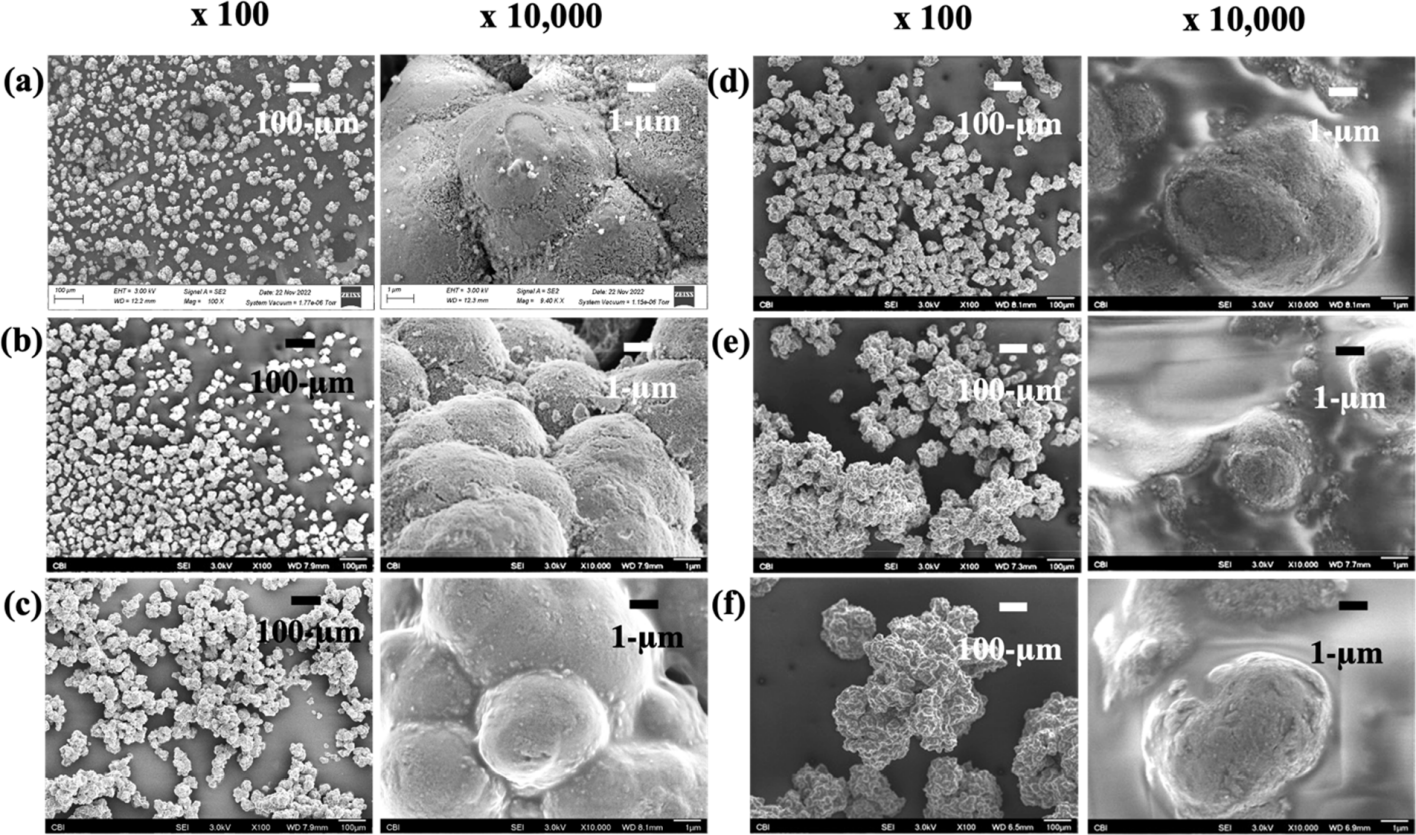
SEM images of uncoated ZrP (a), ZrP-T (b), ZrP-PDMS (c), ZrP-FOTS_1%_ (d), ZrP-FOTS_4%_ (e), and ZrP-FOTS_7%_ (f) at 100× and 10000× magnification. 100× images captured the size distribution and heterogeneity of each material. 10000× identified the reticulated surface of uncoated ZrP versus the other coated versions of ZrP. The exposed, exchangeable surface area diminishes when aggregate size increases within a particle that is coated.

SEMs in Figure 2 of uncoated ZrP, ZrP-T, ZrP-PDMS, ZrP-FOTS_1%_, ZrP-FOTS_4%_, and ZrP-FOTS_7%_ showed the heterogeneity, size distribution, and degree of coating for each material. ZrP-T coated with TEOS had a slightly more irregular and continuous appearance compared to uncoated ZrP. ZrP-PDMS aggregates were approximately 375% larger than uncoated ZrP. The FOTS-coated ZrP size increased with FOTS concentration from 1% (45 ± 5 μm) to 4% (63 ± 10 μm) to 7% (170 ± 79 μm). The ZrP-FOTS_1%_ surface texture was similar to uncoated ZrP. The ZrP-FOTS_4%_ size was 40% larger than ZrP-FOTS_1%_. The surface of ZrP-FOTS_4%_ appeared completely covered with FOTS, and the reticulated ZrP texture was not visible. The ZrP-FOTS_7%_ size was 170% larger than ZrP-FOTS_4%_.

**Figure 3:**
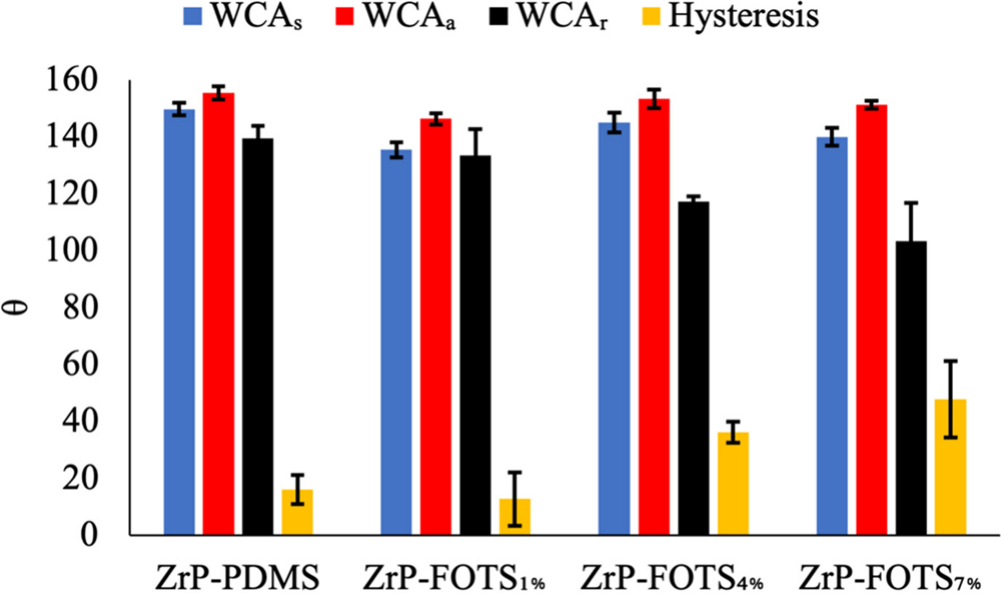
WCA for PDMS- and FOTS-coated ZrP materials. Each experiment was performed three times (*n* = 3). Uncoated ZrP does not have a measurable WCA.

WCA studies investigated WCA_s_, WCA_a_, WCA_r_, and hysteresis for ZrP-PDMS, ZrP-FOTS_1%_, ZrP-FOTS_4%_, and ZrP-FOTS_7%_. The results are given in Figure 3. ZrP-FOTS_1%_ had the lowest WCA_s_ and WCA_a_ of 135.6 ± 3.4° and 138.1 ± 2.7°, respectively. The material’s WCA_a_ was the only one less than 150°. ZrP-FOTS_4%_ had a WCA_s_ of 145.0 ± 3.2° and a WCA_a_ of 153.5 ± 1.5°. ZrP-PDMS WCA_s_ and WCA_a_ were similar to ZrP-FOTS_4%_, though the hysteresis for ZrP-PDMS was nearly 50% less due to lower receding angles on the FOTS-coated structure.

### Competitive Ion Study

**Figure 4:**
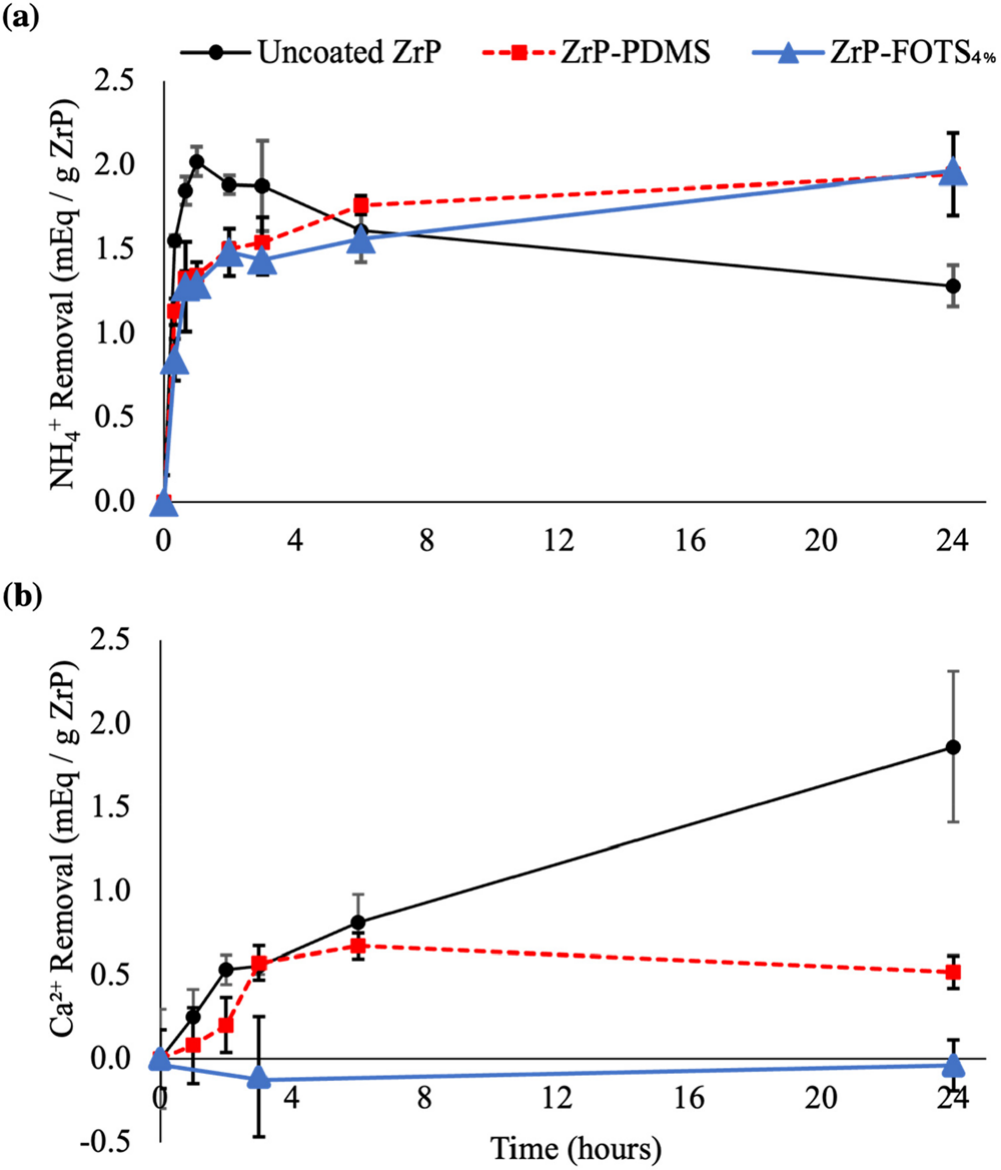
Competitive ion study. Uncoated ZrP, ZrP-PDMS, and ZrP-FOTS_4%_ in 35 mM NH_4_^+^, 35 mM Ca^2^, and 20 mM HEPES water solution. NH_4_^+^ removal in (a) and Ca^2+^ removal in (b). Each experiment was performed three times (*n* = 3). ZrP-PDMS and ZrP-FOTS_4%_ both removed 52% and 53% more NH_4_^+^ than uncoated ZrP by 24 h, respectively. ZrP-PDMS adsorbed 72% less Ca^2+^ then uncoated ZrP while ZrP-FOTS_4%_ did not adsorb any Ca^2+^.

Figure 4 provides the results of the *in vitro* competitive ion study for uncoated ZrP, ZrP-PDMS, and ZrP-FOTS_4%_. The results of ZrP-FOTS 1%, 7%, and 10% are available in Figures S1 and S2 including WCA results. Uncoated ZrP quickly removed 2.02 ± 0.09 mequiv of NH_4_^+^/g of ZrP within the first hour of the study from the testing solution. But by 24 h, uncoated ZrP’s NH_4_^+^ removal decreased by 37% to 1.28 ± 0.12. ZrP-PDMS removed 1.35 ± 0.08 within the first hour and continued removing NH_4_^+^ throughout 24 h to reach 1.94 ± 0.24. ZrP-PDMS increased total NH_4_^+^ removal of ZrP by 52% (1.94 vs 1.28, *p* < 0.05) versus uncoated ZrP at the 24 h time point. ZrP-FOTS_4%_ removed 1.29 ± 0.02 within the first hour. The result had no meaningful statistical difference from ZrP-PDMS (*p* = 0.15). ZrP-FOTS_4%_ removed 1.96 ± 0.02 by the 24 h mark—a 53% increase over uncoated ZrP (*p* < 0.05) but no meaningful statistical difference from ZrP-PDMS (*p* = 0.45).

Uncoated ZrP removed 1.86 ± 0.45 mequiv of Ca^2+^/g of ZrP from the testing solution by the end of the 24 h test. ZrP-PDMS reduced Ca^2+^ removal by 72% (*p* < 0.05) compared to uncoated ZrP. ZrP-FOTS_4%_ and higher FOTS concentrations did not remove any Ca^2+^ throughout the test. The uncoated ZrP selectivity ratio for NH_4_^+^ at 24 h was 0.64. The ZrP-PDMS selectivity ratio for NH_4_^+^ was 3.73 and was 5.4 times greater than uncoated ZrP (*p* < 0.05). ZrP-FOTS_4%_ showed complete selectivity for NH_4_^+^ as no Ca^2+^ removal was detected (*p* < 0.05).

### Acid Exposure Study

**Figure 5:**
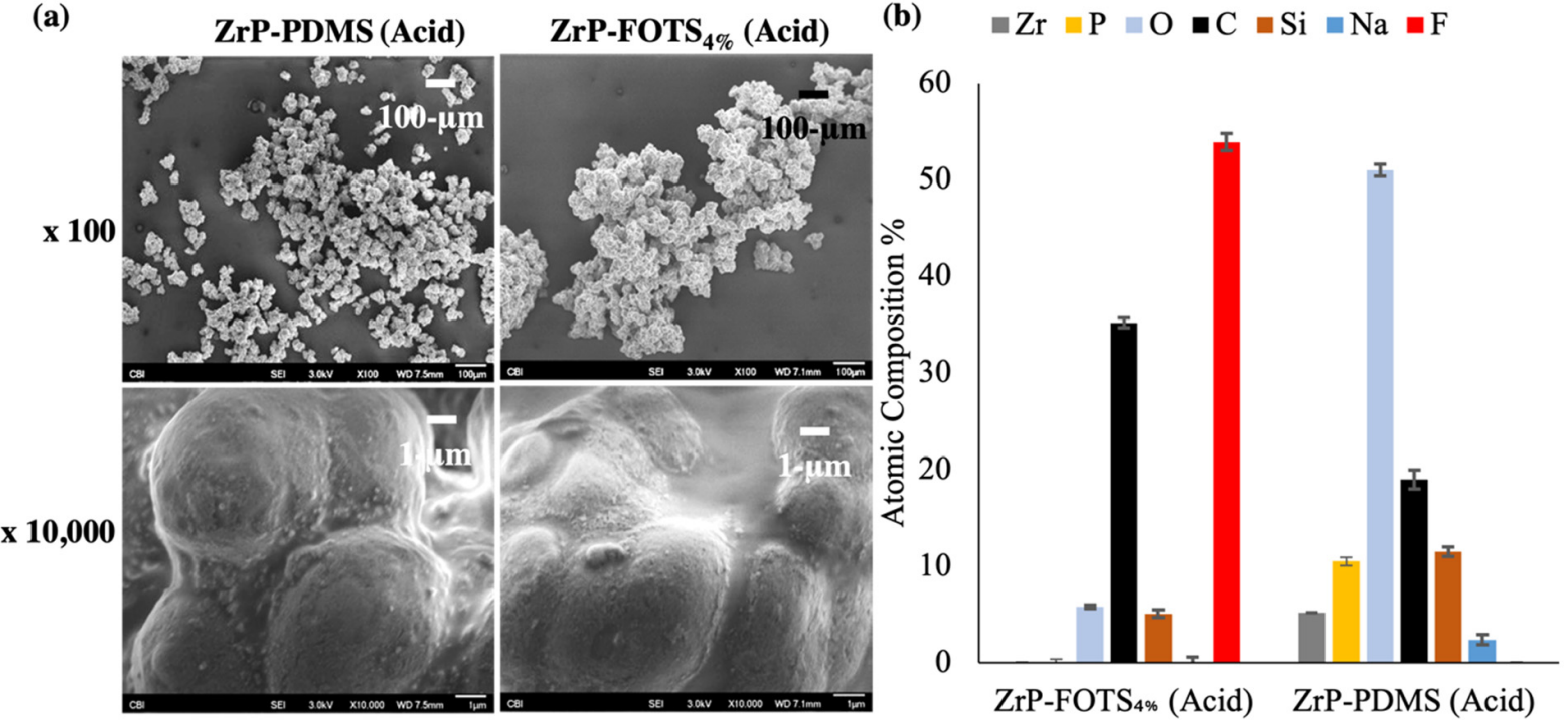
SEM images (a) and XPS results (b) for ZrP-PDMS and ZrP-FOTS_4%_ after exposure to hydrochloric acid (pH = 2.5 ± 0.5) for 3 h. SEM images for both materials were visually similar to the images before acid exposure. XPS results indicated the PDMS coating may have been degraded after acid exposure while the FOTS results were similar to the results before acid exposure. Each XPS analysis was performed three times (*n* = 3).

SEM images and WCA results were collected of ZrP-PDMS and ZrP-FOTS_4%_ materials after acid exposure and are given in Figure 5. SEM images after the acid treatment at 10000× showed both ZrP-PDMS and ZrP-FOTS_4%_ still coated with their respective coatings. The materials were visually similar to the SEM and WCA results before acid exposure (Figures 2 and 3). The 100× zoom appeared to show changes in size of the PDMS and FOTS-coated materials. XPS results in Figure 5b show no change to ZrP-FOTS_4%_ versus Figure 1. However, the PDMS layer appeared to be affected by acid treatment. ZrP-PDMS after acid exposure results appeared to be more similar to uncoated ZrP than to ZrP-PDMS with increasing Zr and P composition. The WCA results in Figure S3 indicated the materials maintained their degree of hydrophobicity with no large decreases observed.

**Figure 6:**
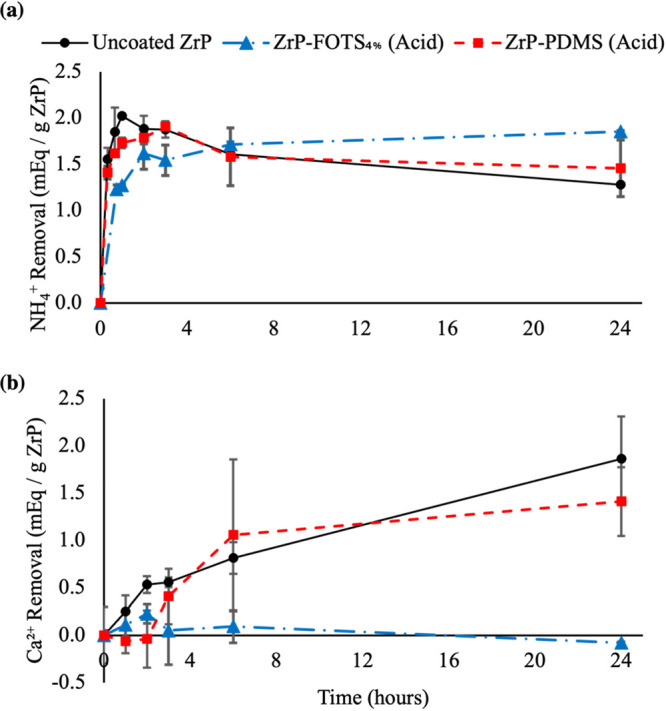
NH_4_^+^ (a) and Ca^2+^ (b) removal over 24 h by ZrP-PDMS and ZrP-FOTS_4%_ after acid exposure. The materials were exposed to HCl_(aq)_ (pH = 1.8) for 3 h to replicate stomach acid conditions. The ion solution was identical with the solution used for removal studies before acid exposure in Figure 4. Each experiment was performed three times (*n* = 3). The results showed ZrP-FOTS_4%_ maintained its selectivity for NH_4_^+^ and removal capacity while ZrP-PDMS selectivity decreased by approximately 72%.

Figure 6 provides the results of the *in vitro* competitive ion study for acid-coated ZrP-PDMS and ZrP-FOTS_4%_ relative to uncoated ZrP. Uncoated ZrP removed 2.02 ± 0.09 within the first hour. Acid-exposed ZrP-PDMS removed 1.73 ± 0.05 mequiv of NH_4_^+^/g of ZrP within the first hour of testing and had a 30% increase in total removal versus ZrP-PDMS before acid exposure. Acid-exposed ZrP-PDMS’ total NH_4_^+^ removal at 24 h decreased by nearly 25% to 1.46 ± 0.31 versus unexposed ZrP-PDMS (*p* < 0.05). The difference in NH_4_^+^ removal between uncoated ZrP and acid-exposed ZrP-PDMS was not statistically significant (*p* = 0.21). Acid-exposed ZrP-FOTS_4%_ removed 1.27 ± 0.01 within the first hour of the study. The total removal at the same time point by unexposed ZrP-FOTS_4%_ was very similar (1.27). Acid-exposed ZrP-FOTS_4%_ continued removing NH_4_^+^ from solution throughout the test and 1.85 ± 0.01 by the end of the study. The result was a 5% decrease from unexposed ZrP-FOTS_4%_ (*p* < 0.05). Acid-exposed ZrP-FOTS_4%_ removed 27% more NH_4_^+^ than acid-exposed ZrP-PDMS (*p* < 0.05) and 46% more than uncoated ZrP (*p* < 0.05) within the study.

Acid-exposed ZrP-PDMS removed 1.41 ± 0.36 mequiv of Ca^2+^/g of ZrP total over the time course of the study. The result was an increase in total Ca^2+^ removal compared to ZrP-PDMS before acid exposure (*p* < 0.05). Acid-exposed ZrP-PDMS removed a similar amount of Ca^2+^ as uncoated ZrP. ZrP-FOTS_4%_ after acid exposure did not remove any Ca^2+^.

Acid-exposed ZrP-PDMS selectivity for NH_4_^+^ decreased by 72% from 3.73 (unexposed to acid) to 1.04. Acid-exposed ZrP-FOTS_4%_ remained completely selective for NH_4_^+^ over Ca^2+^.

### Effect of Coating Structure on Selectivity and Capacity of NH_4_^+^ Removal

The competing ion studies showed ZrP-FOTS_4%_ capable of removing as much NH_4_^+^ as ZrP-PDMS within a solution of 35 mM NH_4_^+^, 35 mM Ca^2+^, and 20 mM HEPES and a starting pH of 8.0 ± 0.5, but with no removal of Ca^2+^. SEM images of FOTS-coated ZrP showed high FOTS concentration results in larger molecules and better coverage. With increase of coating thickness, selectivity increases because water and ion transfer across the membrane is prevented. But total NH_4_^+^ removal within 24 h may decrease if the membrane is too thick. The selectivity and binding capacity results of the competing ion studies in this work showed there was an optimal % FOTS at 4%. Uncoated ZrP’s NH_4_^+^ removal decreased by 37% from its reported 1 h value (2.02 mequiv/g of ZrP) while the other coated materials did not lose NH_4_^+^. The hydrophobic nature of the PDMS-based coating significantly decreased the abundance of Ca^2+^ near the cation-exchanging surface versus uncoated ZrP. And the FOTS-based coating completely removed Ca^2+^ as a competing ion for ZrP binding sites.

The pH of the *in vitro* studies was not maintained as it would be within the small intestine, and pH decreased approximately a whole unit for ZrP-FOTS_4%_ from start of each binding study to the end. pH decreasing by a unit decreases available NH_3_ by 90%. NH_3_ is in equilibrium with NH_4_^+^, and the ratio of NH_3_:NH_4_^+^ is driven by pH. Coatings completely selective for NH_3_ over other competing ions could also be limited by available NH_3_ by the end of the study. When the coated sorbents remove NH_3_ from a solution of NH_4_^+^, there is a free H^+^ left in solution. Future studies should determine whether or not the capacity for NH_4_^+^ removal by FOTS-coated ZrP could be increased significantly if the pH of the solution is held constant as NH_3_ is removed. Prior studies in simulated small intestinal fluid maintained a pH of about 8 and showed improvement in binding of NH_4_^+^ by coated ZrP.^24^

**Scheme 2:**
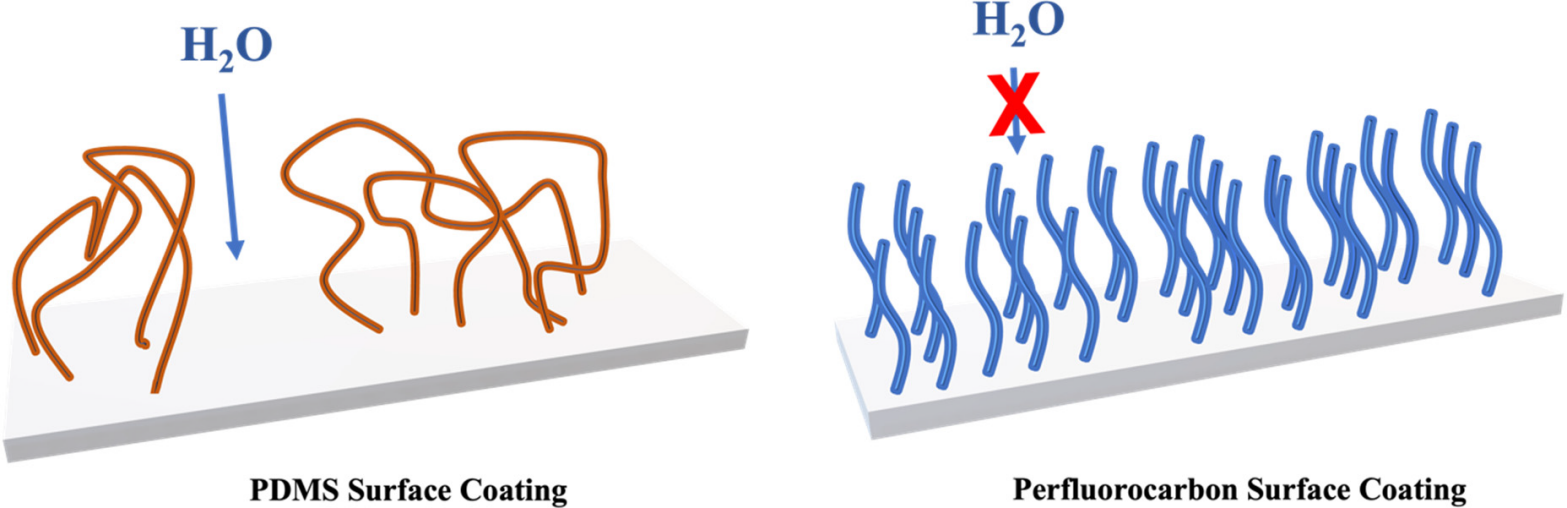
(Left) Flexible PDMS Chains Have Less Orderly Packed Chain Structure; (Right) Rigid PFC Chains Have a More Orderly Packed Chain Structure

PFC-based coatings offer a more rigid chain structure on the surface and were thus selected as the alternative coating material. A more rigid chain would give a more orderly packed chain structure and thus more efficient coverage, which would be impermeable to most small molecules.^35^ In general, a flexible polymer chain is less orderly packed and may not have good coverage. Moreover, higher mobility of flexible polymer chains results in transient dynamic “holes”, i.e., chain-to-chain distance increases due to the motion of polymer segments.^35^ The water solution and ions can then gradually pass through such a membrane and interact with the cation exchanger. PDMS is a flexible polymer by design,^36^ which might explain the imperfect selectivity for NH_4_^+^ in our studies. Scheme 2 illustrates the hypothetical different chain structure between PDMS and PFC coatings.

The more ordered structure of PFC-based coatings could lead to lower NH_3_ permeability compared to PDMS-based coatings,^37^ resulting in lower binding of NH_3_ by the cation exchanger. Increased concentrations of FOTS in coating solution could further hinder total NH_4_^+^ removal capacity by physically covering ion exchange sites on or within the ZrP. The experiments showed ZrP-FOTS_4%_ and ZrP-FOTS_7%_ both achieved complete selectivity for NH_4_^+^. But ZrP-FOTS_4%_ performed better than the other two materials, and ZrP-FOTS_4%_ removed 79% more NH_4_^+^ from solution than ZrP-FOTS_10%_ by the 1 h time point.

PFC’s biological inertness and gas permeability have led to its use in the development of a potential synthetic oxygen-carrier material.^27,28^ FOTS has a reported static WCA of 150° on a substrate with the desired microstructure, categorizing the material as superhydrophobic.^26^

### Effect of Coating Structure on Acid Resistance

Coated ZrP will pass through the stomach after ingestion before entering the small intestine. Low stomach pH could adversely affect the gas-permeable membrane. The pH of the stomach varies between 1 and 5 with a residence time up to 3 h.^38,39^ The pH level of the stomach rises and falls with ingestion of food.^40^ Modified ZrP as an oral sorbent must maintain its function after exposure to stomach acid conditions. The most common pH level within an individual’s stomach is between 1.5 and 2.0.^41,42^ PDMS has been reported to degrade from acid exposure,^43^ while PFC-based coatings like FOTS are known for their chemical inertness and stability.^44^

Acid-treated ZrP-FOTS_4%_ maintained complete NH_4_^+^ selectivity over the 24 h study. But ZrP-PDMS NH_4_^+^ selectivity decreased by over 70% due to acid exposure. XPS results of ZrP-PDMS after acid exposure indicated the PDMS coating may have been degraded. The impact of acid on ZrP-PDMS was apparent with the quick, upfront removal of NH_4_^+^ similar to uncoated ZrP. PDMS degradation could reasonably explain the results for ZrP-PDMS after acid exposure. Acid exposure studies showed a clear advantage for PFC-based coatings over PDMS. A 2013 study assessed PDMS degradation upon exposure to pH values from 2 to 4 and 9 to 12 over multiple days.^43^ The paper showed that silicones can decompose via hydrolysis. The study found that all forms of PDMS will decay to some extent in both alkaline and acidic conditions with −OH-terminated PDMS decaying the most (an order of magnitude greater decay). A degraded PDMS coating may yield an increased rate of water solution reaching the cation exchanger and Ca^2+^ ions along with it. Acid-treated ZrP-FOTS_4%_ adsorption was very similar to the results before acid exposure. One of the attractive features of PFC-based coatings for this application was the known chemical stability and inertness they possess.^44^ FOTS also offers three linking groups to the surface of ZrP per molecule, whereas m-PDMS offers only one group per end. The added groups on FOTS could yield a higher degree of cross-linking and thus stronger resistance to pH degradation. The improved acid resistance of ZrP-FOTS_4%_ versus ZrP-PDMS indicated the PFC-based coatings are a better option as an oral sorbent for ESKD patients.

SEMs of ZrP-FOTS_4%_ indicated FOTS-coated ZrP particles attached to other nearby coated ZrP particles after acid exposure. Acid exposure could have activated unreacted ethoxy groups on FOTS that may have not been activated during the coating process. Though the overall impact on NH_4_^+^ total removal was minimal, future studies could quantify the impact of FOTS-coated ZrP size on removal rate.

The capacity of an oral sorbent for NH_3_ must be as high as possible in order for the product to be tolerated by patients and create clinical benefit. *In vitro* studies of our hydrogen-loaded ZrP have shown a maximum binding capacity for NH_4_^+^ at 4 mequiv/g or more.^45^ The binding of NH_3_ by our coated ZrP products is at most about 1/2 this potential binding capacity. Future studies will investigate the FOTS coating’s NH_4_^+^ removal capacity while maintaining a higher pH throughout the removal process. Additional work should also quantify NH_4_^+^ removal rate by coated ZrP versus uncoated ZrP and assess the material’s function *in vivo*. The technology developed in this work could be relevant to NH_4_^+^ removal in ESKD and liver disease treatment,^46,47^ agricultural management, and wastewater treatment.^20,48^

## Conclusions

This study reports a FOTS-based coating on ZrP with improved NH_4_^+^ removal capacity over uncoated ZrP, complete selectivity for NH_4_^+^ over Ca^2+^, and acid resistance. XPS surface analysis, WCA, and SEM results indicated successful coating of the ZrP surface for both PDMS- and FOTS-based coatings. PDMS-coated ZrP did not maintain its selectivity after acid exposure, but FOTS did. The results showed that ZrP-FOTS_4%_ maintained its selectivity and capacity while ZrP-PDMS selectivity decreased by 72%. ZrP-FOTS_4%_’s complete selectivity for NH_4_^+^ and pH resistance were key improvements over ZrP-PDMS. The more rigid and orderly packed PFC-based chain may have diminished water penetrating the coating. The binding results indicated that optimized FOTS coverage, i.e., ZrP-FOTS_4%_, offered both complete selectivity for NH_4_^+^ in the presence of Ca^2+^ and improved total capacity by nearly 25% over ZrP-FOTS_7%_.

## Supporting Information Available

Figure S1: WCA study for all materials tested before acid exposure; Figure S2: competitive ion study—ZrP-FOTS at 4%, 7%, and 10% and ZrP-F_2_ (no TEOS layer before adding FOTS); Figure S3: surface atomic composition of ZrP-FOTS_4%_ and ZrP-FOTS_7%_ after HCl_(aq)_ (pH = 1.8) exposure for 3 h (PDF)

## Abbreviations

ESKD: end-stage kidney disease
CKD: chronic kidney disease
BUN: blood urea nitrogen
m-PDMS: methoxy-terminated polydimethylsiloxane
TEOS: tetraethyl orthosilicate
XPS: X-ray photoelectron spectroscopy
SEM: scanning electron microscopy
WCA: water contact angle.
