# Supplementary material for: A Perfluorocarbon-Coated ZrP Cation Exchanger with Excellent Ammonium Selectivity and Chemical Stability: An Oral Sorbent for End-Stage Kidney Disease (ESKD)
Source: Langmuir. 2023 May 25;39(22):7912–21. doi: 10.1021/acs.langmuir.3c00753 (PMC10249412; doi:10.1021/acs.langmuir.3c00753)
Supplement: Supplementary file 1 — la3c00753_si_001.pdf [file la3c00753_si_001.pdf]

A Perfluorocarbon-Coated ZrP Cation Exchanger with Excellent Ammonium Selectivity and Chemical Stability: An Oral Sorbent for End-Stage Kidney Disease (ESKD)

Evan Richards[1], Sang-Ho Ye[2,3], Stephen R. Ash[4], Lei Li[1]**

[1] Department of Chemical and Petroleum Engineering, University of Pittsburgh, Pittsburgh PA, 15260

[2] McGowan Institute for Regenerative Medicine, Pittsburgh PA, 15219

[3] Department of Surgery, University of Pittsburgh, Pittsburgh PA, 15260

[4] CEO, HemoCleanse Technologies, LLC, Lafayette IN, 47904

Corresponding Authors:

Stephen R. Ash (Email: sash@hemocleanse.com); Lei Li (Email: lel55@pitt.edu)

Supporting Information

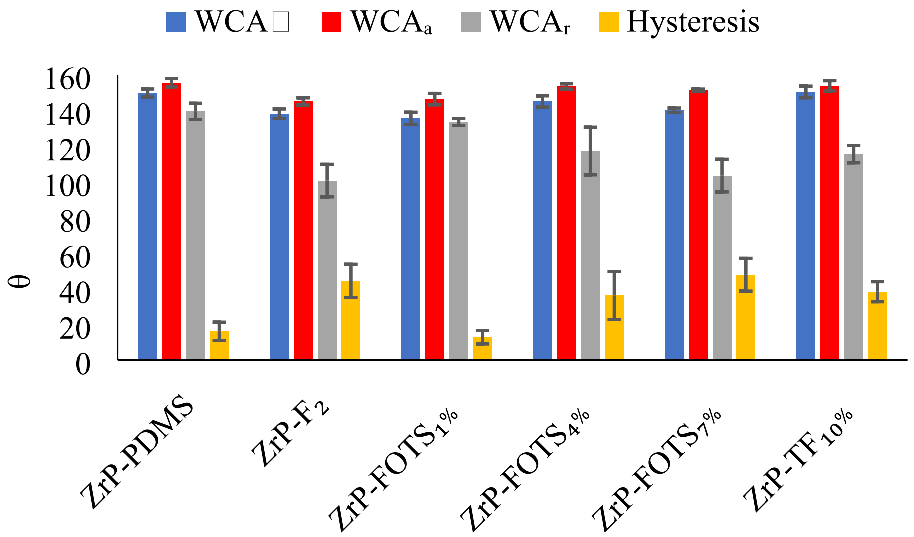

Figure S1. Static, advancing, and receding WCA study of all materials tested before acid exposure.

ZrP-F_2 had similar WCA results to the materials with a polysiloxane-formed membrane on ZrP.

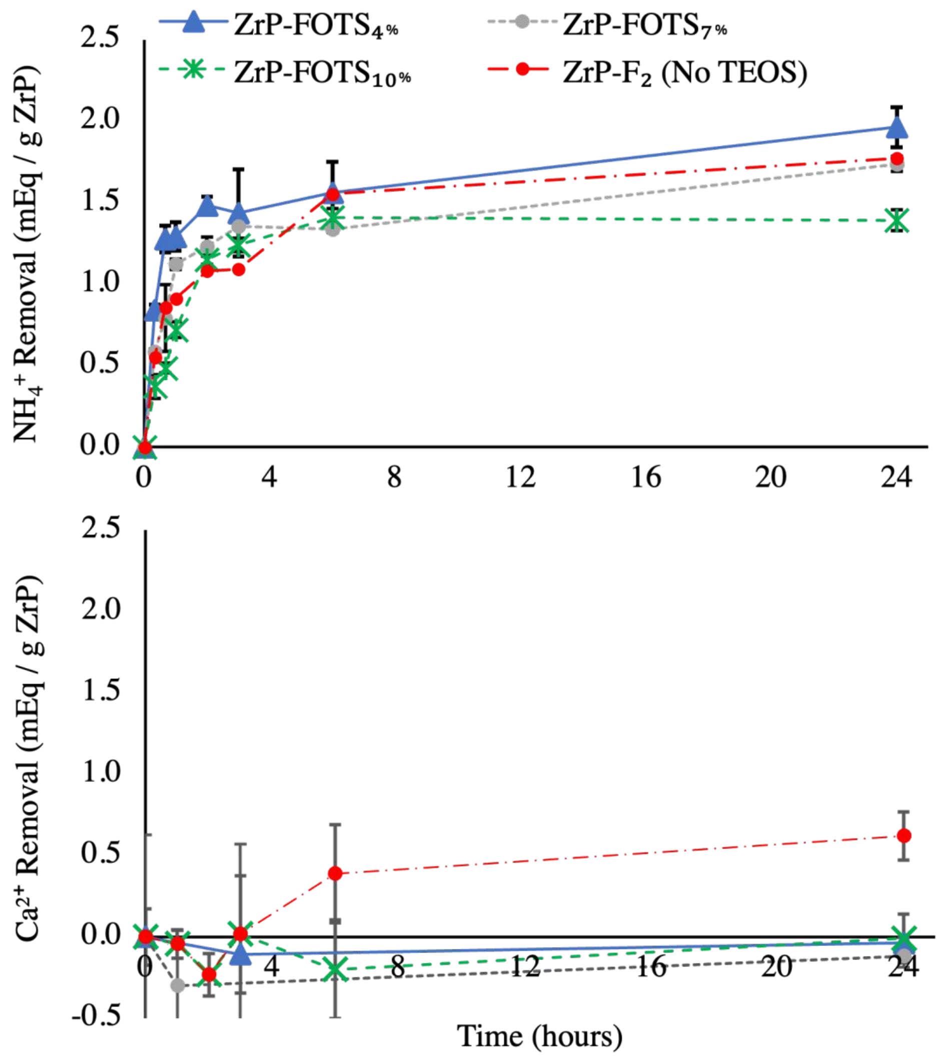

Figure S2. Competitive ion study – ZrP-FOTS at 4%, 7%, and 10% and ZrP-F_2 (No TEOS layer before adding FOTS). 35-mM NH_4^+ and 35-mM Ca^{2+}. NH_4^+ removal in (a) and Ca^{2+} removal in (b). Assessing ZrP-$FOTS_{4\%}$ versus ZrP-F_2 showed the necessity to first coat the material with a polysiloxane membrane for optimal NH_4^+ selectivity. ZrP-F_2 selectivity was nearly three times better than uncoated ZrP, but ultimately did not achieve the same degree of selectivity as ZrP-$FOTS_{4\%}$. ZrP-F_2 also removed 33% less NH_4^+ than both ZrP-PDMS and ZrP-$FOTS_{4\%}$ by the 1

hour timepoint. The polysiloxane membrane increased the abundance of available -OH groups for the gas-permeable and hydrophobic monomers to attach to.

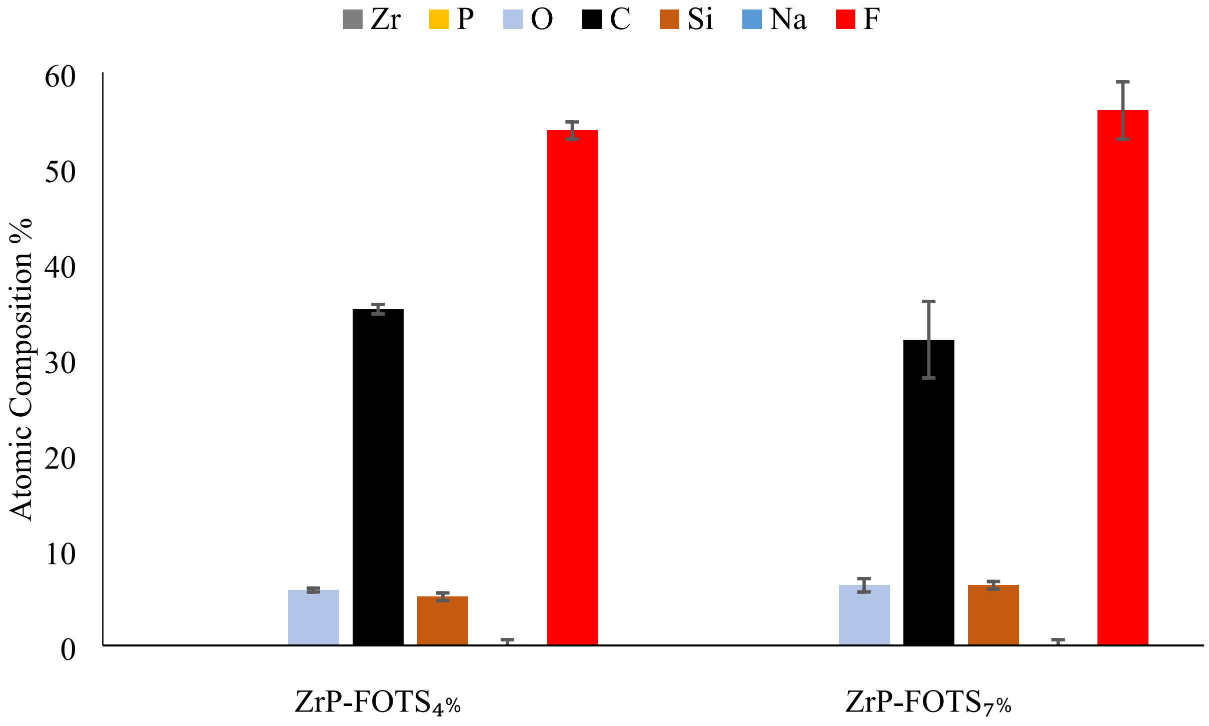

Figure S3. Surface atomic composition of ZrP-FOTS$_{4\%}$ and ZrP-FOTS$_{7\%}$ after $HCl_{(aq)}$ (pH=1.8) exposure for 3 hours. The results indicate minimal change to the surface composition relative to the coatings before acid exposure. All testing was carried out on three separate preparations of coated product.
